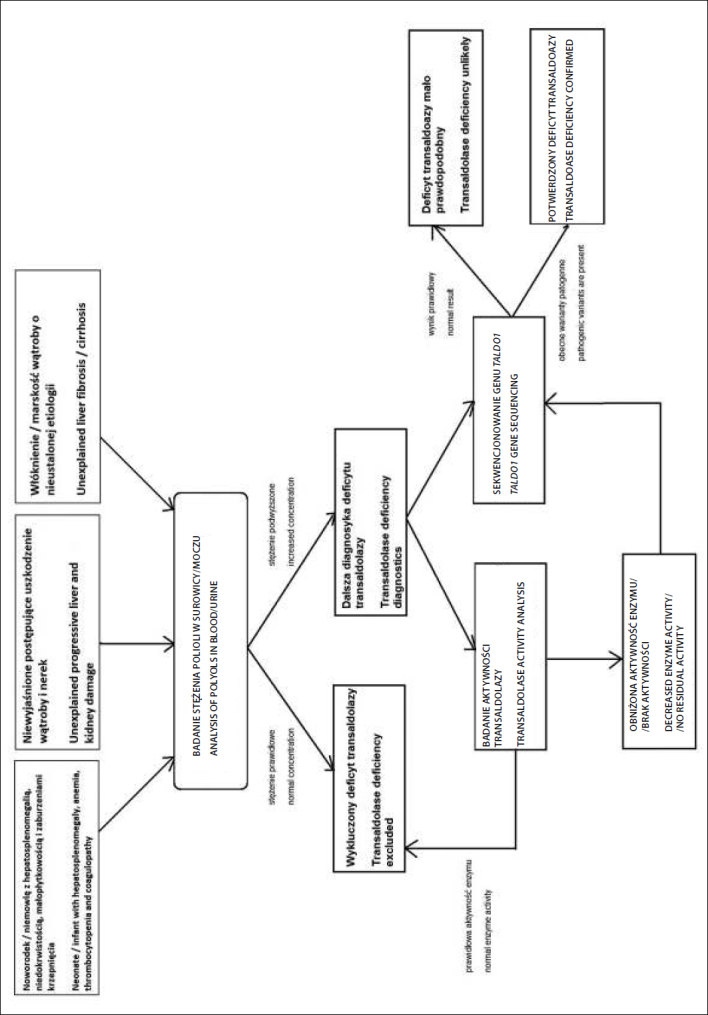# Deficyt Aktywności Transaldolazy – Obraz Kliniczny, Patogeneza, Diagnostyka

**DOI:** 10.34763/devperiodmed.20182202.187196

**Published:** 2018-06-30

**Authors:** Patryk Lipiński, Teresa Stradomska, Anna Tylki-Szymańska

**Affiliations:** 1 Klinika Gastroenterologii, Hepatologii, Zaburzeń Odżywiania i Pediatrii, Instytut ,,Pomnik-Centrum Zdrowia Dziecka’’, Warszawa, Polska; 2 Pracownia Badań Radioimmunologicznych i Biochemii, Instytut ,,Pomnik-Centrum Zdrowia Dziecka’’, Warszawa, Polska; 3 Klinika Pediatrii, Żywienia i Chorób Metabolicznych, Instytut ,,Pomnik-Centrum Zdrowia Dziecka’’, Warszawa, Polska

**Keywords:** transaldolaza, deficyt transaldolazy, szlak przemiany pentoz, poliole, transaldolase, transadolase deficiency, pentose phosphate pathway, polyols

## Abstract

Deficyt aktywności transaldolazy należy do wrodzonych błędów metabolizmu na szlaku przemiany pentoz, który do tej pory rozpoznano i opisano u 33 pacjentów, w tym 4 z Polski.

W artykule przedstawiono obraz kliniczny, patogenezę i diagnostykę choroby. Autorzy przedstawili ponadto własną propozycję algorytmu diagnostyki deficytu transaldolazy.

## Abstract (en)

Transaldolase deficiency is a rare inborn autosomal recessive error of the pentose phosphate pathway that, to date, has been diagnosed in 33 patients, including 4 from Poland.

The aim of this manuscript was to present the clinical presentation, pathogenesis and diagnostic process of transaldolase deficiency. The authors also present a diagnostic algorithm of transaldolase deficiency.

## 1 Wstęp

Deficyt aktywności transaldolazy (ang. *transaldolase deficiency*, TALDO) należy do wrodzonych błędów metabolizmu na szlaku przemiany pentoz (ang. *pentose phosphate pathway*, PPP). Szlak pentozofosforanowy obejmuje ciąg reakcji biochemicznych, zachodzących w cytozolu, których produktami końcowymi są rybozo-5-fosforan i NADPH. Sumaryczne równanie reakcji cyklu pentozofosforanowego jest następujące:

glukozo-6-fosforan + 2NADP^+^ + H_2_O → rybozo-5-fosforan + 2NADPH + 2H^+^ + CO_2_.

Ta droga przemian biochemicznych spełnia dwie role:

- wytwarzanie NADPH (zredukowany dinukleotyd nikotynoamidoadeninowy), który spełnia rolę donora protonów i elektronów w redukcyjnych procesach biosyntezy.
- dostarczanie reszt rybozy do biosystezy nukleotydowi kwasów nukleinowych.

Szczególną intensywność wykazuje on w okresie wzrastania płodu oraz w pierwszych latach życia [1, 2, 3].

Przemiany na szlaku pentozofosforanowym dzielimy na dwie fazy: nieodwracalną fazę oksydacyjną i odwracalną fazę nieoksydacyjną. Transaldolaza (EC 2.2.1.2), obok transketolazy, jest enzymem odwracalnej fazy cyklu pentozofosforanowego i katalizuje następującą reakcję:

sedoheptulozo-7-fosforan + gliceraldehydo-3-fosforan → erytrozo-4-fosforan + fruktozo-6-fosforan

W wyniku deficytu transaldolazy dochodzi do nagromadzenia polioli – erytritolu, arabitolu, rybitolu, sedoheptitolu, perseitolu oraz siedmiowęglowych cukrów − sedoheptulozy, mannoheptulozy i fosfosedoheptulozy.

Celem pracy jest analiza obrazu klinicznego, patogenezy i diagnostyki deficytu aktywności transaldolazy na podstawie danych z piśmiennictwa oraz doświadczeń własnych. Jak do tej pory, w dostępnych publikacjach nie został przedstawiony algorytm diagnostyczny, stąd autorzy przedstawili propozycję własną algorytmu diagnostyki TALDO.

## 2 Obraz kliniczny

Deficyt aktywności transaldolazy został po raz pierwszy opisany przez Verhoeven i wsp., w 2001 r., u kilkumiesięcznego niemowlęcia z wewnątrzmacicznym opóźnieniem wzrastania, hepatosplenomegalią i zaburzeniami krzepnięcia [4]. Biopsja wątroby, wykonana w 2. roku życia, wykazała drobnoguzkową marskość wątroby. W wieku kilkunastu lat, opisywany pacjent, rozwinął przewlekłą niewydolność wątroby i tubulopatię [4, 5].

Dotychczas, TALDO opisano u 33 pacjentów (tabela I**)**, w tym 4 z Polski [4-18]. Większość (79%) z nich pochodziła od spokrewnionych rodziców, w tym 12 z jednego arabskiego rodu [11].

**Tabela I:** Charakterystyka pacjentów z deficytem transaldolazy. Table I. Characteristics of TALDO deficient patients reported in the literature.

Pacjent *Patient*	Manifestacja prenatana *Antenatal* *manifestation*	Obraz kliniczny *Clinical outcome*	Wiek w momencie rozpoznania *Age at diagnosis*	Wiek ostatniego badania *Age at last follow-up*	badania Wynik molekularnego *Molecular variant* */protein effect*	Piśmiennictwo *References*
1	lUGR	marskość wątroby, tubulopatia, przewlekła choroba nerek, zgon w wieku 17 lat *liver cirrhosis, tubulopathy, chronic kidney failure, died at 17 years of age*	10 lat *10 years*	17 lat *17 years*	c.512_514delCCT/ *p.Serl71del; hmz*	Verhoeven et al [4]
2	zespól HELLP *HELLP syndrome*	niewydolność wątroby, niewydolność *tubulopatia*, krążeniowo-*zgon w 18*. oddechowa, *dobie życia liver failure, respiratory failure, heart failure, tubulopathy, died at 18 days of age*	*post mortem* (diagnoza pośmiertna) *post mortem diagnosis*	18 dni *18 days*	c.575G>A/ *p.Argl92His; hmz*	Verhoeven et al [6]
3	znaczny (20 kg) przyrost masy ciała matki w ciąży *excessive maternal weight gain (20 kg) during pregnancy*	zgon w wieku 5 miesięcy *died at 5 months of age*	*post mortem* (diagnoza pośmiertna) *post mortem diagnosis*	5 miesięcy 5 *months*	c.512_514delCCT/ *p.Serl71del; hmz*	Valayannopoulos et al [7]
4	obrzęk płodowy *hydrops fetalis*	terminach ciąży *termination of pregnancy*	(diagnoza *post mortem* pośmiertna) *post mortem diagnosis*			
5	prawidłowy rozwój *normal*	marskość wątroby, tubulopatia, przewlekła choroba nerek *liver cirrhosis, tubulopathy, chronic kidney failure*	4 lata *4 years*	9 lat *9 years*		
6	prawidłowy rozwój *normal*	marskość wątroby, tubulopatia *liver cirrhosis, tubulopathy*	3 miesiące *3 months*	4 lata 10 miesięcy *4 years 10 months*		
7	matowodzie, splenomegalia *oligohydramnios, splenomegaly*	marskość wątroby, tubulopatia *liver cirrhosis, tubulopathy*	*2* lata 2 *years*	*2* lata 2 *years*	C.574C>T/ *p.Argl92Cys; hmz*	Wamelink et al [9]
8	prawidłowy rozwój *normal*	wielonarządowa niewydolność, zgon w wieku 4.5 miesięcy *multiorgan failure, died at 4.5 months of age*	*post mortem* (diagnoza pośmiertna) *post mortem diagnosis*	4.5 miesięcy *4.5 months*	c.895_897delAAC/ p.Asn299del; *c. 931 G>A/ p. Gly311Arg; comphtz*	Balasubramaniam et al [8]
9	prawidłowy rozwój *normal*	rak wątrobowokomórkowy, przeszczepienie wątroby *hepatocellular carcinoma, liver transplantation*	*1 rok* *1 year*	16 miesięcy *16 months*	C.512C>T/ *p.Ser171Phe; hmz*	LeDuc et al [12]
10	prawidłowy rozwój *normal*	marskość wątroby *liver cirrhosis*	3 lata *3 years*	3 lata *3 years*		
11	prawidłowy rozwój *normal*	prawidłowy rozwój normal	8 lat *8 years*	9 lat *9 years*		
12	prawidłowy rozwój *normal*	marskość wątroby *liver cirrhosis*	10 lat *10 years*	10 lat *10 years*	c.793delC/ p.Gln265Argfs*56 *; hmz* et Eyaid al [11], Jassim et al [14]	
13	wielowodzie *polyhydramnios*	zgon w wieku 5 miesięcy *died at 5 months of age*	*post mortem* (diagnoza pośmiertna) *post mortem diagnosis*	5 miesięcy *5 months*		
14	prawidłowy rozwój *normal*	hepatomegalia *hepatomegaly*	6 miesięcy *6 months*	6 miesięcy *6 months*		
15	prawidłowy rozwój *normal*	marskość wątroby *liver cirrhosis*	4 lata *4 years*	4 lata *4 years*		
16	prawidłowy rozwój *normal*	hepatosplenomegalia, pancytopenia *hepatosplenomegaly, pancytopenia*	2.5 lat 2.5 *years*	2.5 lat *2.5 years*		
17	hiperechogenny kardiomegalia, obraz jelit *cardiomegaly, hyperechogenic bowel on ultrasound*	zespół marskość wątrobowo-wątroby, płucny *liver cirrhosis, hepatopulmonary syndrome*	1.5 lat *1.5 years*	1.5 lat *1.5 years*		
18	prawidłowy rozwój *normal*	marskość wątroby *liver cirrhosis*	5 lat *5 years*	5 lat *5 years*		
19	małowodzie, IUGR, całkowite odwrócenie trzew, hepatosplenomegalia *IUGR, oligohydramnios, situs inversus totalis, hepatosplenomegaly*	marskość wątroby, zaburzenia krzepnięcia, małopłytkowość, neutropenia *liver cirrhosis, neutropenia, thrombocytopaenia, bleeding diathesis*	12 miesięcy *12 months*	12 miesięcy *12 months*	c.793delC/ p.Gln265Argfs*56; hmz Eyaid et al [11], et Jassim al [14]	
20	prawidłowy rozwój *nomal*	nepatomegalia, naczyniak wątroby	b.d. *not known*	b.d. *not known*		
21	IUGR	hepatosplenomegalia *hepatosplenomegaly*	8 miesięcy 8 months	8 miesięcy *8 months*		
22	prawidłowy rozwój *normal*	hepatosplenomegalia, złóg w nerkach w wieku 7 ms *hepatosplenomegaly, renal calculus observed at 7 months of age*	2.5 lat 2.5 *years*	2.5 lat 2.5 *years*		
23	prawidłowy rozwój *normal*	hepatosplenomegalia, małopłytkowość *hepatosplenomegaly, thrombocytopenia*	7 lat 7 *years*	7 lat 7 *years*		
24	b.d. *not known*	hepatosplenomegalia, podwyższona aktywność aminotransferaz, prawidłowy koagulogram *hepatosplenomegaly, elevated serum transaminases, normal coagulation profile*	*2* lata 2 *years*	*2* lata 2 *years*	C.574C>T/ *p.Arg192Cys; hmz* Al-Shamsi et al [15]	
25	b.d. *not known*	hepatomegalia, małopłytkowość, prawidłowa aktywność aminotransferaz, prawidłowy koagulogram, rybia łuska, zez, wtórny brak miesiączki *hepatomegaly, thrombocytopenia, normal serum transaminases, normal coagulation profile, ichthyosis, nystagmus, secondary amenorrhoea*	25 lat 25 *years*	25 lat 25 *years*		
26	b.d. *not known*	przeszczepienie wątroby (w 1. roku życia), obecnie podwyższona aktywność aminotransferaz, prawidłowy koagulogram *liver transplantation (at 1 year of age), currently normal coagulation profile and slightly elevated serum transaminases*	*1 rok* *1 year*	3 lata *3 years*	C.574C>T/ *p.Arg192Cys; hmz* Al-Shamsi et al [15]	
27	obrzęk płodowy *hydrops fetalis*	niewydolność oddechowa wtórna do krwotoku płucnego *severe respiratory distress due to pulmonary haemorrhage*	1 miesiąc *1 month*	3 miesiące *3 months*		
28	lUGR, hiperechogenny obraz jelit *IUGR, hyperechogenic bowel on ultrasound*	niedrożność smółkowa jelit, niewydolność wątroby, zgon w wieku 2 miesięcy *meconium plug, liver failure, died at months of age*	*post mortem* (diagnoza pośmiertna) *post mortem diagnosis*	*2* miesiące 2 *months*	C.669C>G/ p.Tyr223*hmz	Banne et al [16]
29	prawidłowy rozwój *normal*	hepatomegalia, podwyższona aktywność aminotransferaz, prawidłowy koagulogram *hepatomegaly, elevated liver transaminases, normal coagulation profile*	9 miesięcy *9 months*	16 miesięcy *16 months*	C.574C>T/ *p.Argl92Cys*	Rodan et al [17]
30	znaczny (20 kg) przyrost masy ciała matki w ciąży, IUGR *excessive maternal weight gain (20 kg) during pregnancy, IUGR*	marskość wątroby, tubulopatia *liver cirrhosis, tubulopathy*	3.5 lat *3.5 years*	13 lat *13 years*	c.575G>A/ *p.Argl92His; hmz*	Tylki-Szymańska et al [10,13], Lipiński et al [18]
31	znaczny (20 kg) przyrost masy ciała matki w ciąży, IUGR *excessive maternal weight gain (20 kg), IUGR*	marskość wątroby, tubulopatia *liver cirrhosis, tubulopathy*	6 miesięcy *6 months*	10 lat *10 years*	c.575G>A/ *p.Arg192His; hmz*	
32	małowodzie, IUGR *oligohydramnios, IUGR*	marskość wątroby, tubulopatia, kamica nerkowa *liver cirrhosis, tubulopathy, nephrolithiasis*	*2* miesiące 2 *months*	8 lat *8 years*	c.462-174_981+53del/p.?; *hmz*	
33	obrzęk płodowy, IUGR *hydrops fetalis, IUGR*	włóknienie wątroby, tubulopatia, kamica nerkowa *liver fibrosis, tubulopathy, nephrolithiasis*	1.5 lat *1.5 years*	4 lata *4 years*	c.575G>A/p.Argl92His; *c. 462-174 981 +53del/ p. ?; comphtz*	
comphtz – złożona heterozygota; hmz – homozygota; b.d. – brak danych; IUGR – wewnątrzmaciczne zahamowanie rozwoju *comphtz – compound heterozygote; hmz – homozygote; IUGR – intrauterine growth retardation*						

Obraz kliniczny TALDO jest zróżnicowany, jednak zawsze obejmuje postępujące uszkodzenie wątroby oraz nerek (dysfunkcja cewek nerkowych) [5]. Wyróżnia się postać o wczesnym początku (ang. *early-onset TALDO*) – wystąpienie objawów w okresie prenatalnym lub w ciągu pierwszych 3 miesięcy życia, stanowiącą chorobę o ciężkim przebiegu i niepomyślnym rokowaniu – oraz postać objawiającą się później (ang. *late-onset TALDO*) – powyżej 3. miesiąca życia, stanowiącą powoli postępującą chorobę z możliwym długoletnim przeżyciem [18].

Większość przypadków opisanych w literaturze stanowiło TALDO o wczesnym początku. U noworodków i niemowląt stwierdzano małą masę urodzeniową – odnotowywano już wewnątrzmaciczne opóźnienie wzrastania, powiększenie wątroby i śledziony, niedokrwistość, małopłytkowość, zaburzenia krzepnięcia, a w kilku przypadkach obrzęk płodowy. W niektórych przypadkach przebieg ciąży charakteryzował się masywnym przyrostem masy ciała u matek oraz dużym i nieprawidłowym łożyskiem. Blisko połowa tych pacjentów zmarła w okresie niemowlęcym, najczęściej w wyniku krwawienia, jako efektu koagulopatii w przebiegu uszkodzenia wątroby [4, 5, 6, 7, 8, 9, 10, 11, 12, 13, 14, 15, 16, 17, 18].

Nieco odmiennie przedstawia się przebieg deficytu transaldolazy o wczesnym początku u polskich pacjentów, którzy mimo początkowo złego rokowania (masywne wodobrzusze, hipoalbuminemia wymagająca okresowych wlewów albumin), wkroczyli w drugą dekadę życia (3 z 4 pacjentów) z objawami skompensowanej marskości wątroby i kontrolowanej tubulopatii (poprzez leczenie substytucyjne) [18].

TALDO o późnym początku wystąpienia objawów, mimo potencjalnie lepszego rokowania, prowadzi powoli do postępującego uszkodzenia wątroby i nerek. LeDuc i wsp. opisali 8-letniego chłopca, u którego TALDO zostało wykryte przypadkowo (skrining rodzinny) [12]. Al-Shamsi i wsp. opisali TALDO u 25-letniej pacjentki, która w dzieciństwie (brak dokładnych danych) prezentowała hepatosplenomegalię i małopłytkowość, a rozpoznanie TALDO postawiono przypadkowo w toku diagnostyki wtórnego braku miesiączki [15].

W przebiegu TALDO w wątrobie stwierdza się charakterystyczne drobnoguzkowe włóknienie, a ostatecznie marskość wątroby. Z czasem u chorych z dłuższym okresem przeżycia pojawiają się objawy tubulopatii – hiperkalciuria, białkomocz cewkowy (kłębuszkowo-cewkowy) – zwykle jako pierwsze objawy, aminoaciduria, glukozuria, kwasica kanalikowa – oraz stopniowo rozwija się przewlekła choroba nerek [5]. W obrazie klinicznym stwierdza się ponadto osteopenię/osteoporozę oraz niskorosłość (skutki tubulopatii i uszkodzenia wątroby).

Charakterystyczne dla TALDO są zmiany skórne – poszerzona siatka naczyń i teleangiektazje na skórze tułowia (zwłaszcza pleców) oraz tendencja do tworzenia naczyniaków jamistych (*haemangioma cavernosum*). W literaturze znajdują się ponadto opisy pacjentów z *cutis laxa* oraz hipertrychozą [4, 5, 6, 7, 8, 9, 10, 11, 12, 13, 14, 15, 16, 17, 18].

Ponadto występują zaburzenia endokrynologiczne − u ok. 1/3 wszystkich pacjentów w okresie niemowlęcym stwierdzano nieprawidłowości w zakresie zewnętrznych narządów płciowych (wnętrostwo, kliteromegalia), a badania hormonalne wykazały u części z nich hipogonadyzm hipergonadotropowy.

W niektórych przypadkach opisywane są wrodzone wady serca, do najczęstszych należą drożny przewód tętniczy oraz ubytek przegrody międzyprzedsionkowej [4, 5, 6, 7, 8, 9, 10, 11, 12, 13, 14, 15, 16, 17, 18]. Część autorów opisuje cechy dysmorfii, czego nie stwierdzono u polskich pacjentów, jednakże należy mieć na uwadze, że cechy te nie zostały dokładnie sprecyzowane i powinny być podobne w opisywanych przypadkach.

Rozwój psychoruchowy i umysłowy pacjentów są prawidłowe [4, 5, 6, 7, 8, 9, 10, 11, 12, 13, 14, 15, 16, 17, 18].

## 3 Patogeneza, diagnostyka

Deficyt aktywności transaldolazy prowadzi do kumulacji w płynach ustrojowych polioli − erytritolu, arabitolu, rybitolu, sedoheptitolu, perseitolu i siedmiowęglowych cukrów − sedoheptulozy, mannoheptulozy i fosfosedoheptulozy [2, 19, 20, 21, 22].

Diagnostyka opiera się na stwierdzeniu wydalania z moczem, jak i kumulacji w surowicy (metodą spektrometrii mas) rybitolu, arabitolu, erytritolu, oraz o najwyższej czułości sedoheptulozy i sedoheptulozo-7-fosforanu [2, 19, 20, 21, 22]. Wstępna metoda diagnostyczna (stosowana w IP-CZD), opiera się określeniu stosunku D-/L- arabinitolu w moczu (normy przedstawiono w tabeli II [22]).

**Tabela II:** Poziom D-/L-arabinitolu w moczu w zależności od wieku [22]. Table II. Urinary D-/L-arabitol ratio depending on age [22].

Wiek [lata] *Age [years]*	D-/L-Aarabinitol *D-/L- arabinitol*
0-1	<3,6
>1-3	<3,3
>3-10	<3,0
>10	<2,5

Potwierdzenie rozpoznania wymaga badania aktywności transaldolazy w leukocytach krwi obwodowej lub hodowanych fibroblastach skóry. Metodę weryfikacji rozpoznania może stanowić również badanie molekularne. Badania enzymatyczne mogą być pominięte na rzecz badań molekularnych (sekwencjonowanie genu *TALDO1*) (rycina 1).

W patogenezie schorzenia podkreśla się toksyczny efekt działania gromadzonych metabolitów (ufosforylowanych cukrów) na hepatocyty i kanaliki nerkowe. Inna hipoteza podkreśla znaczenie stresu oksydacyjnego, który jest efektem braku NADPH koniecznego dla odtwarzania zredukowanej formy glutationu – kofaktora peroksydazy glutationowej przeciwdziałającej stresowi oksydacyjnemu [2, 4]. Reduktaza glutationowa, tworząca układ antyoksydacyjny w mitochondriach, także wykorzystuje NADPH jako źródło elektronów, katalizując reakcję odtwarzania zredukowanej formy glutationu kosztem utlenienia NADPH. Tym samym zapewnia równowagę peroksydacyjno-antyoksydacyjną, zapobiegając stresowi oksydacyjnemu.

## 4 Leczenie

Leczenie pacjentów z TALDO jest wyłącznie objawowe. Upośledzenie czynności syntetycznej wątroby może wiązać się z koniecznością okresowej podaży albumin czy osoczowych czynników krzepnięcia. Podobnie, upośledzenie czynności cewek nerkowych może wymagać suplementacji nieorganicznych fosforanów, podaży alfa-kalcydiolu czy wyrównywania zaburzeń elektrolitowych.

Dyskusyjne są wskazania do ewentualnego przeszczepienia wątroby. Niektórzy autorzy sugerują możliwość uszkodzenia przeszczepionej wątroby [3, 4, 5, 6, 7, 8, 9, 10, 11, 12, 13, 14, 15, 16]. W dwóch opublikowanych przypadkach dokonano przeszczepienia wątroby w okresie niemowlęcym, jednakże przed rozpoznaniem TALDO dotychczas nie opublikowano efektów terapeutycznych.

Przeszczepienie wątroby u polskich pacjentów wobec względnie stabilnego ich stanu i równoczesnego uszkodzenia nerek nie jest obecnie brane pod uwagę [17]. Ze względu na rzadkość występowania, wskazane jest monitorowanie i prowadzenie pacjentów z TALDO w jednym, mającym doświadczenie oraz odpowiednie zasoby do diagnostyki, ośrodku.

## 5 Podsumowanie

U pacjentów z niewyjaśnionym postępującym uszkodzeniem wątroby i nerek wskazane jest wykonanie badania kumulacji polioli. Potwierdzenia/wykluczenia choroby wymaga również każdy przypadek noworodka/niemowlęcia z hepatosplenomegalią, niedokrwistością, małopłytkowością i zaburzeniami krzepnięcia, zwłaszcza urodzonego z małą urodzeniową masą ciała (oraz dużym przyrostem masy ciała matki w trakcie trwania ciąży).

Włóknienie, marskość wątroby o nieustalonej etiologii zawsze wymaga badania stężenia polioli.

**Figure:**